# 4-(2-Hydroxy­benzyl­idene­amino)­benzonitrile

**DOI:** 10.1107/S160053680801564X

**Published:** 2008-06-07

**Authors:** Xing-Xuan Gong, Hai-Jun Xu

**Affiliations:** a Ordered Matter Science Research Center, College of Chemistry and Chemical Engineering, Southeast University, Nanjing 210096, People’s Republic of China

## Abstract

The mol­ecule of the title compound, C_14_H_10_N_2_O, is nearly planar. There is a strong intra­molecular O—H⋯N hydrogen bond between the imine and hydr­oxy groups. The configuration with respect to the C=N double bond is *anti* (1*E*).

## Related literature

For related literature, see: Allen *et al.* (1987 ▶); Chen *et al.* (2008 ▶); Cheng *et al.* (2005 ▶, 2006 ▶); Elmah *et al.* (1999 ▶); May *et al.* (2004 ▶); Weber *et al.* (2007 ▶); Xu *et al.* (2008 ▶).
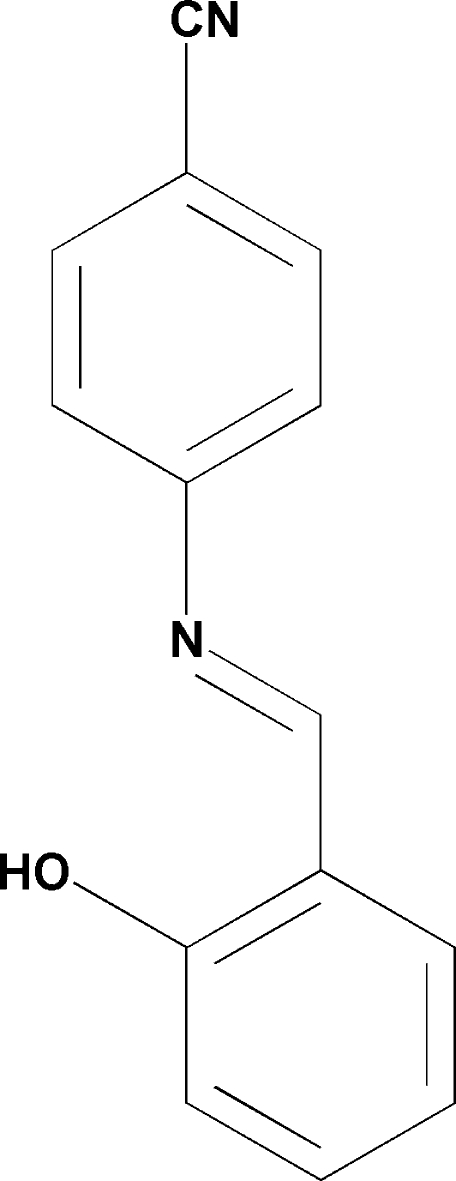


## Experimental

### 

#### Crystal data

C_14_H_10_N_2_O
*M* *_r_* = 222.24
Monoclinic, 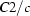

*a* = 28.071 (6) Å
*b* = 5.8471 (12) Å
*c* = 14.687 (3) Å
β = 109.91 (3)°
*V* = 2266.6 (9) Å^3^
*Z* = 8
Mo *K*α radiation
μ = 0.08 mm^−1^
*T* = 293 (2) K
0.12 × 0.11 × 0.03 mm

#### Data collection

Rigaku Mercury2 diffractometer
Absorption correction: multi-scan (*CrystalClear*; Rigaku, 2005 ▶) *T*_min_ = 0.915, *T*_max_ = 1.00 (expected range = 0.913–0.997)
9782 measured reflections
2223 independent reflections
971 reflections with *I* > 2σ(*I*)
*R*_int_ = 0.132

#### Refinement

*R*[*F* ^2^ > 2σ(*F* ^2^)] = 0.077
*wR*(*F* ^2^) = 0.176
*S* = 0.97
2223 reflections
154 parameters
H-atom parameters constrained
Δρ_max_ = 0.18 e Å^−3^
Δρ_min_ = −0.18 e Å^−3^

### 

Data collection: *CrystalClear* (Rigaku, 2005 ▶); cell refinement: *CrystalClear*; data reduction: *CrystalClear*; program(s) used to solve structure: *SHELXS97* (Sheldrick, 2008 ▶); program(s) used to refine structure: *SHELXL97* (Sheldrick, 2008 ▶); molecular graphics: *SHELXTL* (Sheldrick, 2008 ▶); software used to prepare material for publication: *SHELXTL*.

## Supplementary Material

Crystal structure: contains datablocks I, global. DOI: 10.1107/S160053680801564X/dn2347sup1.cif

Structure factors: contains datablocks I. DOI: 10.1107/S160053680801564X/dn2347Isup2.hkl

Additional supplementary materials:  crystallographic information; 3D view; checkCIF report

## Figures and Tables

**Table 1:** Hydrogen-bond geometry (Å, °)

*D*—H⋯*A*	*D*—H	H⋯*A*	*D*⋯*A*	*D*—H⋯*A*
O1—H1*B*⋯N1	0.82	1.88	2.609 (4)	147

**Fig. 1.**
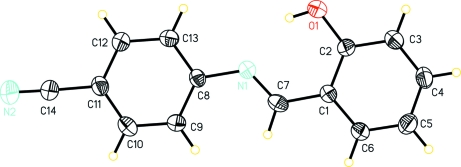
A view of the title compound with the atomic numbering scheme. Displacement ellipsoids are drawn at the 30% probability level. H atoms are represented as small spheres of arbitrary radii.

## supplementary crystallographic information

### Comment

The Schiff base compounds have received considerable attention for several decades, primarily due to their importance in the development of coordination chemistry related to magnetism (Weber *et al.*, 2007), catalysis (Chen *et al.*, 2008) and biological process (May *et al.*,2004). Recently, we have reported a Schiff base compound (Xu *et al.*, 2008). As an extention of our work on the structural characterization of Schiff base compounds, the title compound has been synthesized.

The molecule of the title compound is nearly planar, the two aromatic rings are only twisted by a dihedral angle  3.28 (18) ° (Fig. 1), As expected, the molecule displays a *trans* configuration about the central C7=N1 imine double bond. Bond lengths and bond angles in the compound are within normal ranges (Allen *et al.*, 1987). The C7=N1 bond length of 1.280 (4) Å indicates a high degree of double-bond character comparable with the corresponding bond lengths in other Schiff bases (1.280 (2) Å; Elmah *et al.*, 1999).

A strong O–H···N intramolecular hydrogen-bond interaction is observed in the molecular structure (Table 1 ) as also found in previous reports (Xu *et al., *2008; Cheng *et al.*, 2006, 2005).

### Experimental

All chemicals were obtained from commercial sources and used without further purification except for salicylaldehyde which is distilled under reduced pressure before use. 4-aminobenzonitrile (1.18 g, 10 mmol) and salicylaldehyde (1.22 g, 10 mmol) were dissolved in ethanol (20 ml). The mixture was heated to reflux for 4 h, then cooled to room temperature overnight then large amounts of a yellow precipitate were formed. Yellow crystals were obtained by recrystallization from ethyl alcohol(yield: 81%). ^1^H-NMR(CDCl_3_, 300 MHz): δ6.98 (t, 1 H), 7.04 (d, 1 H), 7.34(d, 2 H), 7.43 (t, 2 H), 7.72 (d, 2H), 8.61 (s, 1 H). ^13^C-NMR (CDCl_3_)δ110.1, 117.4, 118.6, 118.7, 119.4, 122.1, 132.8, 133.5, 134.3, 152.4, 161.2, 165.0. Esi-MS: calcd for C_14_H_9_N_2_O – H *m*/*z *221.24, found 221.34. Suitable single crystals of the title compound  were obtained after one week by slow evaporation from an ethyl alcohol solution.

### Refinement

All H atoms attached to C atoms and O atom were fixed geometrically and treated as riding with C—H = 0.93 °H (C) and O-H= 0.82 (1)Å with U_iso_(H) = 1.2U_eq_(C) or U_iso_(H) = 1.5U_eq_(O).

### Crystal data

**Table:**

C_14_H_10_N_2_O	*F*_000_ = 928
*M**_r_* = 222.24	*D*_x_ = 1.303 Mg m^−^^3^
Monoclinic, *C*2/*c*	Mo *K*α radiation λ = 0.71073 Å
Hall symbol: -C 2yc	Cell parameters from 7031 reflections
*a* = 28.071 (6) Å	θ = 3.1–29.0º
*b* = 5.8471 (12) Å	µ = 0.08 mm^−^^1^
*c* = 14.687 (3) Å	*T* = 293 (2) K
β = 109.91 (3)º	Block, yellow
*V* = 2266.6 (9) Å^3^	0.12 × 0.11 × 0.03 mm
*Z* = 8	

### Data collection

**Table:**

Rigaku Mercury2 (2x2 bin mode) diffractometer	2223 independent reflections
Radiation source: fine-focus sealed tube	971 reflections with *I* > 2σ(*I*)
Monochromator: graphite	*R*_int_ = 0.132
Detector resolution: 13.6612 pixels mm^-1^	θ_max_ = 26.0º
*T* = 293(2) K	θ_min_ = 3.5º
ω scans	*h* = −34→34
Absorption correction: multi-scan(CrystalClear; Rigaku, 2005)	*k* = −7→7
*T*_min_ = 0.915, *T*_max_ = 1.00	*l* = −18→18
9782 measured reflections	

### Refinement

**Table:**

Refinement on *F*^2^	Secondary atom site location: difference Fourier map
Least-squares matrix: full	Hydrogen site location: inferred from neighbouring sites
*R*[*F*^2^ > 2σ(*F*^2^)] = 0.077	H-atom parameters constrained
*wR*(*F*^2^) = 0.176	*w* = 1/[σ^2^(*F*_o_^2^) + (0.0584*P*)^2^] where *P* = (*F*_o_^2^ + 2*F*_c_^2^)/3
*S* = 0.97	(Δ/σ)_max_ < 0.001
2223 reflections	Δρ_max_ = 0.18 e Å^−^^3^
154 parameters	Δρ_min_ = −0.18 e Å^−^^3^
Primary atom site location: structure-invariant direct methods	Extinction correction: none

### Special details

**Table:**

Geometry. All e.s.d.'s (except the e.s.d. in the dihedral angle between two l.s. planes) are estimated using the full covariance matrix. The cell e.s.d.'s are taken into account individually in the estimation of e.s.d.'s in distances, angles and torsion angles; correlations between e.s.d.'s in cell parameters are only used when they are defined by crystal symmetry. An approximate (isotropic) treatment of cell e.s.d.'s is used for estimating e.s.d.'s involving l.s. planes.
Refinement. Refinement of *F*^2^ against ALL reflections. The weighted *R*-factor *wR* and goodness of fit *S* are based on *F*^2^, conventional *R*-factors *R* are based on *F*, with *F* set to zero for negative *F*^2^. The threshold expression of *F*^2^ > σ(*F*^2^) is used only for calculating *R*-factors(gt) *etc*. and is not relevant to the choice of reflections for refinement. *R*-factors based on *F*^2^ are statistically about twice as large as those based on *F*, and *R*- factors based on ALL data will be even larger.

### Fractional atomic coordinates and isotropic or equivalent isotropic displacement parameters (Å^2^)

**Table:**

	*x*	*y*	*z*	*U*_iso_*/*U*_eq_	
C13	0.34671 (13)	0.0317 (6)	0.9348 (3)	0.0487 (10)	
H14A	0.3362	−0.0983	0.9592	0.058*	
C7	0.22581 (13)	0.3041 (6)	0.8582 (2)	0.0466 (10)	
H13A	0.2331	0.4471	0.8385	0.056*	
N1	0.26153 (10)	0.1571 (4)	0.8889 (2)	0.0443 (8)	
C3	0.11223 (12)	0.0032 (6)	0.8792 (3)	0.0504 (10)	
H12A	0.1038	−0.1343	0.9017	0.061*	
C10	0.37793 (13)	0.4177 (6)	0.8639 (3)	0.0527 (11)	
H11A	0.3889	0.5492	0.8414	0.063*	
C11	0.41195 (13)	0.2453 (7)	0.9055 (3)	0.0479 (10)	
C8	0.31195 (12)	0.1997 (6)	0.8920 (2)	0.0396 (9)	
C9	0.32765 (13)	0.3953 (6)	0.8557 (3)	0.0485 (10)	
H8A	0.3045	0.5095	0.8262	0.058*	
O1	0.19746 (9)	−0.1128 (4)	0.92932 (18)	0.0630 (8)	
H1B	0.2253	−0.0677	0.9302	0.095*	
C2	0.16187 (13)	0.0481 (6)	0.8873 (3)	0.0439 (9)	
C1	0.17471 (12)	0.2547 (6)	0.8532 (2)	0.0384 (9)	
C6	0.13639 (13)	0.4125 (6)	0.8116 (3)	0.0497 (10)	
H5A	0.1444	0.5505	0.7888	0.060*	
C14	0.46381 (14)	0.2676 (6)	0.9108 (3)	0.0591 (12)	
C12	0.39627 (13)	0.0523 (6)	0.9420 (3)	0.0522 (10)	
H3A	0.4193	−0.0625	0.9713	0.063*	
C4	0.07550 (14)	0.1627 (7)	0.8377 (3)	0.0545 (11)	
H2A	0.0422	0.1319	0.8323	0.065*	
C5	0.08702 (14)	0.3697 (7)	0.8034 (3)	0.0602 (12)	
H1A	0.0618	0.4769	0.7754	0.072*	
N2	0.50503 (13)	0.2785 (6)	0.9144 (3)	0.0899 (13)	

### Atomic displacement parameters (Å^2^)

**Table:**

	*U*^11^	*U*^22^	*U*^33^	*U*^12^	*U*^13^	*U*^23^
C13	0.052 (2)	0.041 (2)	0.055 (3)	0.0006 (19)	0.020 (2)	0.0061 (19)
C7	0.058 (3)	0.045 (2)	0.038 (2)	−0.009 (2)	0.0190 (19)	0.0019 (18)
N1	0.0419 (17)	0.0436 (18)	0.048 (2)	−0.0016 (15)	0.0161 (15)	0.0017 (15)
C3	0.051 (2)	0.051 (2)	0.051 (3)	−0.007 (2)	0.019 (2)	0.003 (2)
C10	0.055 (2)	0.052 (2)	0.056 (3)	−0.011 (2)	0.026 (2)	0.001 (2)
C11	0.042 (2)	0.055 (2)	0.049 (2)	−0.005 (2)	0.0189 (19)	0.001 (2)
C8	0.038 (2)	0.046 (2)	0.036 (2)	−0.0043 (18)	0.0143 (17)	−0.0006 (18)
C9	0.049 (2)	0.046 (2)	0.051 (3)	0.0014 (19)	0.0181 (19)	0.0106 (19)
O1	0.0538 (17)	0.0503 (16)	0.087 (2)	0.0003 (13)	0.0273 (15)	0.0197 (14)
C2	0.042 (2)	0.045 (2)	0.044 (2)	0.0030 (19)	0.0135 (19)	0.0047 (18)
C1	0.038 (2)	0.042 (2)	0.035 (2)	−0.0046 (18)	0.0124 (17)	−0.0021 (17)
C6	0.049 (2)	0.050 (2)	0.053 (3)	−0.001 (2)	0.0212 (19)	0.009 (2)
C14	0.045 (3)	0.061 (3)	0.070 (3)	0.002 (2)	0.017 (2)	0.009 (2)
C12	0.044 (2)	0.050 (2)	0.062 (3)	0.0027 (19)	0.017 (2)	0.005 (2)
C4	0.042 (2)	0.069 (3)	0.056 (3)	−0.003 (2)	0.021 (2)	0.003 (2)
C5	0.054 (3)	0.065 (3)	0.063 (3)	0.014 (2)	0.021 (2)	0.010 (2)
N2	0.050 (2)	0.097 (3)	0.124 (4)	0.002 (2)	0.033 (2)	0.029 (3)

### Geometric parameters (Å, °)

**Table:**

C13—C12	1.364 (4)	C11—C14	1.437 (5)
C13—C8	1.376 (4)	C8—C9	1.395 (4)
C13—H14A	0.9300	C9—H8A	0.9300
C7—N1	1.280 (4)	O1—C2	1.358 (4)
C7—C1	1.440 (4)	O1—H1B	0.8200
C7—H13A	0.9300	C2—C1	1.401 (4)
N1—C8	1.422 (4)	C1—C6	1.391 (4)
C3—C4	1.370 (4)	C6—C5	1.373 (4)
C3—C2	1.383 (4)	C6—H5A	0.9300
C3—H12A	0.9300	C14—N2	1.142 (4)
C10—C11	1.380 (5)	C12—H3A	0.9300
C10—C9	1.381 (4)	C4—C5	1.391 (5)
C10—H11A	0.9300	C4—H2A	0.9300
C11—C12	1.384 (4)	C5—H1A	0.9300
			
C12—C13—C8	121.2 (3)	C8—C9—H8A	120.3
C12—C13—H14A	119.4	C2—O1—H1B	109.5
C8—C13—H14A	119.4	O1—C2—C3	118.1 (3)
N1—C7—C1	122.0 (3)	O1—C2—C1	121.4 (3)
N1—C7—H13A	119.0	C3—C2—C1	120.5 (3)
C1—C7—H13A	119.0	C6—C1—C2	118.3 (3)
C7—N1—C8	123.0 (3)	C6—C1—C7	119.8 (3)
C4—C3—C2	119.5 (3)	C2—C1—C7	121.8 (3)
C4—C3—H12A	120.2	C5—C6—C1	121.6 (3)
C2—C3—H12A	120.2	C5—C6—H5A	119.2
C11—C10—C9	120.1 (3)	C1—C6—H5A	119.2
C11—C10—H11A	119.9	N2—C14—C11	178.0 (5)
C9—C10—H11A	119.9	C13—C12—C11	119.5 (3)
C10—C11—C12	120.2 (3)	C13—C12—H3A	120.3
C10—C11—C14	119.5 (3)	C11—C12—H3A	120.3
C12—C11—C14	120.2 (4)	C3—C4—C5	121.4 (3)
C13—C8—C9	119.5 (3)	C3—C4—H2A	119.3
C13—C8—N1	115.6 (3)	C5—C4—H2A	119.3
C9—C8—N1	124.9 (3)	C6—C5—C4	118.6 (3)
C10—C9—C8	119.4 (3)	C6—C5—H1A	120.7
C10—C9—H8A	120.3	C4—C5—H1A	120.7

### Hydrogen-bond geometry (Å, °)

**Table:**

*D*—H···*A*	*D*—H	H···*A*	*D*···*A*	*D*—H···*A*
O1—H1B···N1	0.82	1.88	2.609 (4)	147
